# A near-miss diagnosis of necrotizing breast fasciitis complicated by atrial fibrillation secondary to septic focus: a case report and brief review of literature

**DOI:** 10.1259/bjrcr.20220120

**Published:** 2023-01-30

**Authors:** Rachel Jane Klapper, Benjamin Joseph Michael Horn, Benedict Amalraj, Maamannan Venkataraj, Mohammad Abdurrehman Sheikh, Dominika Pullmann, Kiran Malikayil, Jeffrey Wooliscroft

**Affiliations:** 1 The Department of Internal Medicine, Louisiana State University Health Sciences Center-Shreveport, Shreveport, Louisiana, United States; 2 Department of Radiology, Louisiana State University Health Sciences Center-Shreveport, Shreveport, Louisiana, United States; 3 Department of Surgery, New York Medical College - Metropolitan Hospital Center, New York, New York, United States

## Abstract

Necrotizing fasciitis of the breast is a rare, life-threatening entity characterized by a rapidly aggressive infection of the soft tissue. There are few literature reports on necrotizing fasciitis at the level of the breast tissue as the most common locations are within the abdominal wall or extremities, but this entity can lead to sepsis and systemic multiorgan failure if not adequately managed. Here, we report a case that highlights the course of a 68-year-old African American female with a past medical history of hypertension, hyperlipidemia, and poorly controlled diabetes mellitus, who presented with the complaint of a painful right breast abscess with intermittent, purulent drainage. An initial point-of-care ultrasound displayed an area of induration of the right breast as well as soft tissue edema with no identifiable fluid collection. A subsequent CT abdomen and pelvis was obtained given new onset abdominal pain, which demonstrated incidental findings of inflammatory changes and subcutaneous emphysema along with colonic diverticulosis. Surgical intervention was immediately sought for which she underwent debridement and exploration of the right breast with findings that were consistent with necrotizing transformation. The patient was sent back to the OR for an additional surgical debridement the next day. Of note, the patient had post-op atrial fibrillation with rapid ventricular response and had to be admitted to the ICU for conversion to sinus rhythm. She returned to sinus rhythm and was transferred back to medicine before application of a negative pressure wound dressing on discharge. The patient was transitioned from Enoxaparin to Apixaban for anticoagulation control in the setting of atrial fibrillation before being discharged to a Skilled Nursing Facility with long-term antibiotics. This case highlights the difficulty and significance in establishing a prompt diagnosis for necrotizing fasciitis.

## Introduction

Necrotizing fasciitis (NF) is a rare, aggressive, and life-threatening condition with a significant mortality rate often as high as 73%.^1,2^ It is characterized by a soft tissue infection that has spread to underlying subcutaneous tissue and fascia.^3^ It is predominantly found in the literature to affect the extremities, abdominal wall, or near the anus or vaginal canal, but is infrequently found within the breast, and even then, most cases present following traumatic events or surgical interventions such as with resection.^2^

The initial presentation of necrotizing soft tissue infections varies from a minor infection of the gross soft tissue to severe forms that can present with septic shock or multiorgan system dysfunction and failure. Given the high mortality rate associated with this diagnosis, prompt interventions including intravenous (i.v.) antibiotics and urgent surgical intervention are necessary. Early recognition and appropriate management can reduce mortality by nearly 10%, thus proper diagnosis is paramount.^4,5^ Delayed presentation and intervention can result in significant morbidity and mortality including the need for total resection or even death.

According to Cai et al. in 2021, only 38 cases of NF of the breast have been reported in the literature as of 2021.^3^ A brief review of the literature on PubMed for this year, 2022, only showed four recent cases of NF of the breast. Given a relative scarcity of reports, this case report highlights the rare finding of NF of the breast as well as the subsequent hospital course associated with early intervention.^6^

## Case presentation

The patient is a 68-year-old African American female with a past medical history of hypertension, hyperlipidemia, and poorly controlled diabetes mellitus, who initially presented to the emergency department with a 1-week history of a painful right breast abscess with intermittent, purulent drainage. She also reported symptoms of fever, chills, and decreased oral intake secondary to generalized fatigue and malaise and denied any discharge per nipple, symptoms of the contralateral breast, or weight loss. Family history was negative for known cases of breast cancer. Per patient, an inciting event may have been a mosquito bite to the area 1 week prior.

On initial presentation, the patient was fatigued, but in no acute distress. Her vitals were notable for a heart rate of 98 beats per minute, blood pressure 166/80 mmHg, a temperature of 99.1 F, and an oxygen saturation of 100% on room air. Examination of her right breast revealed an open wound superior to the inframammary fold with thick, foul-smelling drainage. Her labs were significant for leukocytosis of 21 × 10^3^ /microliter, hemoglobin of 11.5 g dl^−1^, CRP elevation to 52.7 mg dl^−1^, creatinine of 1.70 mg dl^−1^, hyperglycemia with a blood glucose of 469 mg dl^−1^, and a sodium of 131 mmol l^−1^.

**Figure 1.**
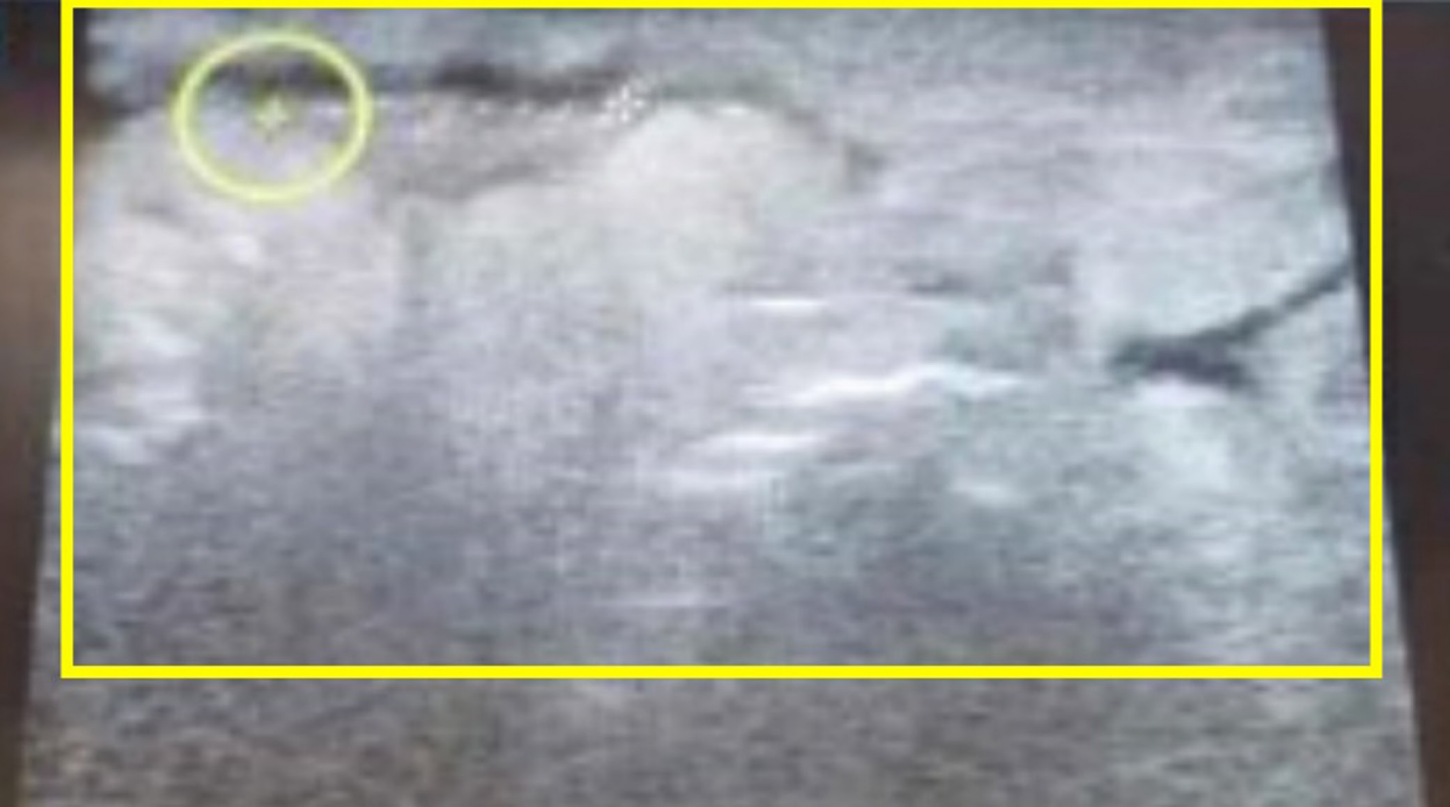
Imaging obtained from a bedside ultrasound machine in the Emergency Department. The yellow box displays an area of induration at the inferior portion of the right breast as well as soft tissue edema with no discrete drainable fluid collection.

Surgery was initially consulted for evaluation of the abscess and possible bedside surgical intervention. The surgical team subsequently performed a bedside ultrasound demonstrating induration at the inferior portion of the right breast as well as soft tissue edema with no discrete drainable fluid collection (Figure 1). No surgical intervention was recommended at the time as the surgical team suspected an infected cyst *vs* an abscess for which the standard of care consists of drainage and antibiotic treatment. As the abscess had already started to drain, cultures were obtained and sent before the patient was admitted for empiric antibiotic therapy. Upon admission, patient received vancomycin 1.5 g daily and piperacillin-tazobactam 3.375 g every 6 h, which was later transitioned to cefepime 2 g bd and metronidazole 500 mg every 8 h given development of an acute kidney injury (AKI).

**Figure 2.**
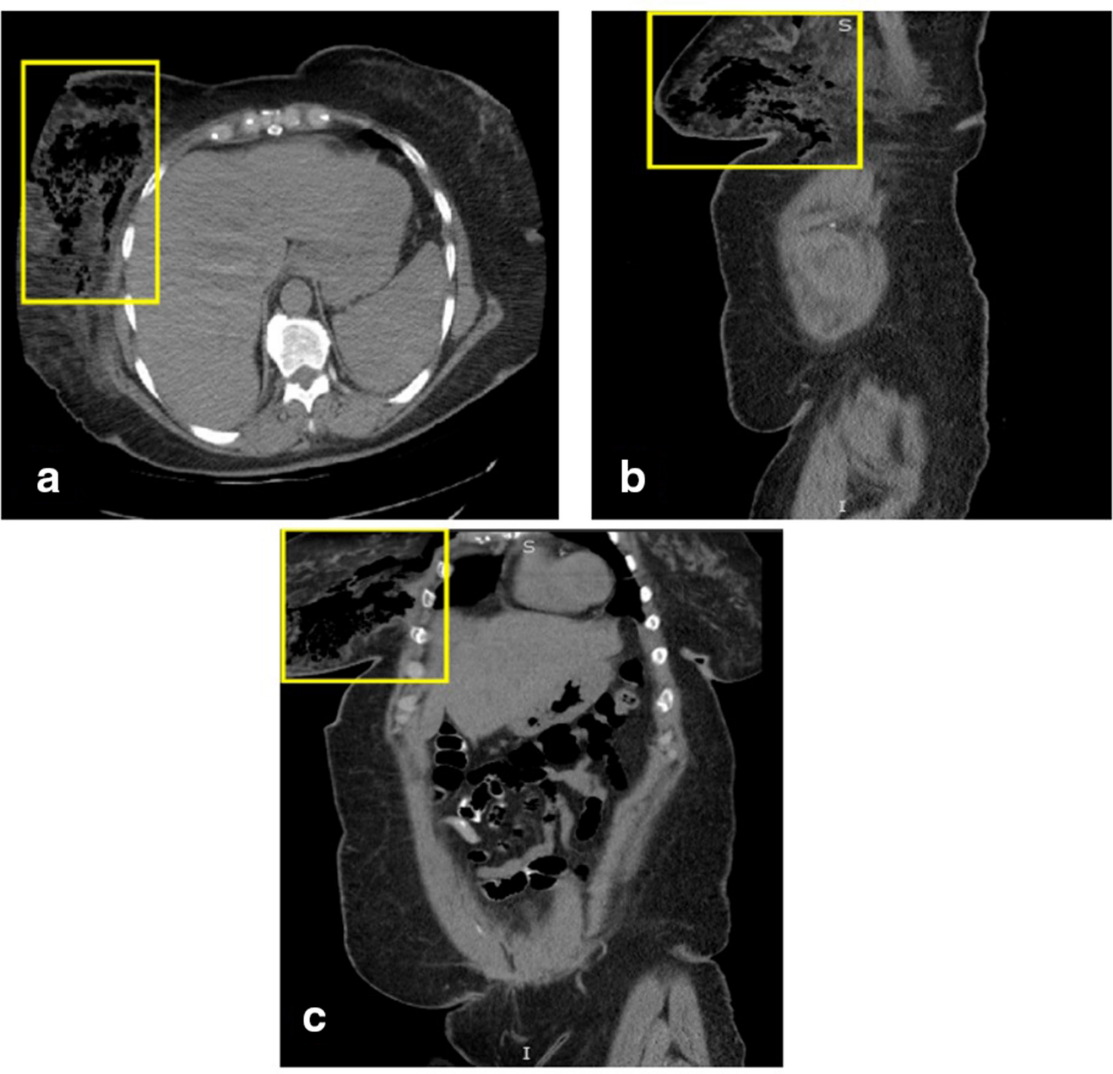
CT imaging displays the reported inflammatory changes and subcutaneous emphysema of the right breast. A displays an axial view with the yellow box demonstrating significant inflammatory changes and subcutaneous emphysema in the right breast. **B** displays a sagittal view with the yellow box highlighting subcutaneous emphysema in the right breast and inflammatory changes. Finally, **C** highlights the coronal view of the CT abdomen pelvis with the yellow box signifying the area of subcutaneous emphysema in the right breast and the abdominal wall.

**Figure 3.**
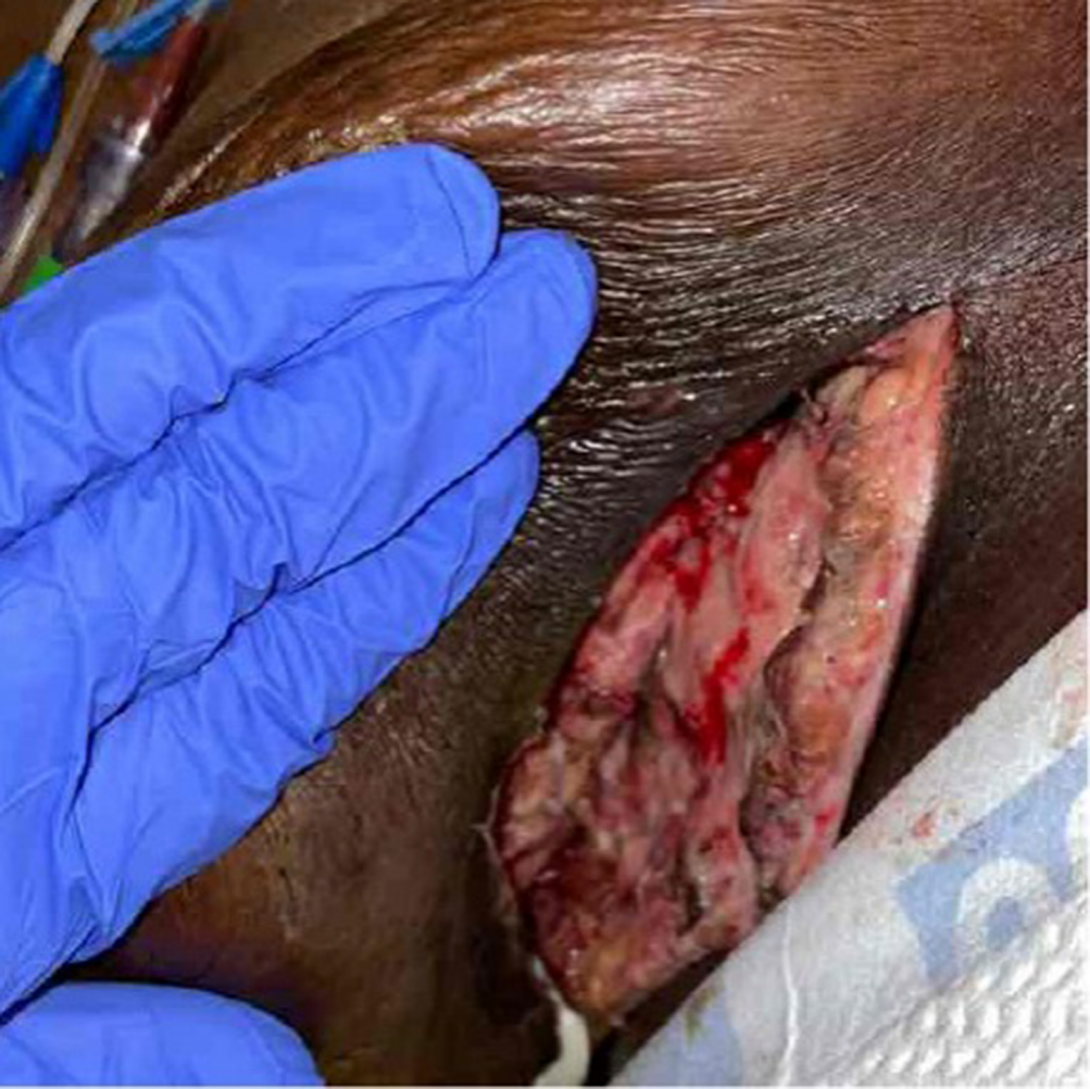
Post-surgical site of the right breast superior to the inframammary fold after debridement of additional necrotic tissue. The tissue is clean with viable tissue appreciable.

On hospital day 2, the patient began to develop new onset sharp, stabbing, generalized abdominal pain. As part of the work-up, CT abdomen pelvis without contrast was obtained as the patient was found to have AKI with a creatinine of 1.6 (baseline 0.9), likely prerenal in nature due to poor oral intake. A CT abdomen pelvis without contrast was ordered to spare further kidney damage. The findings displayed colonic diverticulosis without evidence of diverticulitis. Importantly, an incidental finding was made of significant inflammatory changes and subcutaneous emphysema within the right breast extending to the upper abdominal wall (Figure 2). Given these new radiographic findings and concerns for rapid progression of the infection, there was an immediate need to rule out necrotizing transformation. The patient was subsequently taken to the operating room for urgent surgical exploration and debridement of the right breast. During surgery, the wound was noted to have continued thin, gray, foul-smelling drainage with pockets of purulent fluid extending deeply. A significant amount of necrotic fat and breast tissue was identified; however, sparing the chest wall musculature, consistent with necrotizing soft tissue infection involving the fascia. Accordingly, the area was aggressively debrided, irrigated, tissue specimens were sent for culture and pathology, and the wound was left open with wound packing and a Penrose drain in place. The patient was again taken to the operating room for surgical exploration and additional debridement the following day where approximately 90% of the wound was found to have healthy, viable tissue (Figure 3). On post-operative day 4 of the index surgery, a negative pressure dressing was applied with which the patient was to be discharged home.

Following surgical exploration and debridement, antibiotic coverage was broadened from Piperacillin-Tazobactam, which was discontinued, to Meropenem 1 g and Clindamycin 600 mg every 8 h for necrotizing fasciitis. Vancomycin was continued for MRSA coverage. Cultures grew presumptive Proteus species with gram stain positive for Gram-positive cocci. Infectious disease was consulted for recommendations regarding the patient’s antibiotic regimen, which was continued.

The post-operative course was complicated by atrial fibrillation (AFib) in the Post Anesthesia Care Unit (PACU); no prior cardiac history was noted. Anesthesia administered 5 mg of i.v. Esmolol without return to a normal sinus rhythm. An EKG was ordered demonstrating AFib with rapid ventricular response at a rate of 135–150 beats per minute. A bedside chest X-ray was obtained to confirm proper placement of the central line placed previously in the operating room. The catheter tip inserted via the right internal jugular vein was noted to project over the area of the superior atriocaval junction. Accordingly, the catheter was retracted slightly with repeat imaging showing the tip now in the superior vena cava at the level of T6-7. Medicine was consulted and recommended repeat dosing with 5 mg i.v. Metoprolol, which was given, however, with no change in rhythm. Normal sinus rhythm was finally achieved after two additional doses of Metoprolol 5 mg i.v. were given. Cardiology was consulted for further evaluation and found that the arrhythmia may have been secondary to sepsis. Following the PACU, the patient was transferred to the critical care unit for monitoring and was started on 25 mg Metoprolol three times daily (TID). She was placed on anticoagulation with Enoxaparin 40 mg subcutaneously until being transitioned to Apixaban 5 mg two times daily (BID) on discharge. Follow- up was performed by daily wound checks by general surgery; no additional imaging was performed of the surgical site. Prior to discharge, the patient was found to be clinically improving and the antibiotic regimen was subsequently narrowed to Cefazolin 2 g i.v. every 8 h and Metronidazole 500 mg TID. The patient was discharged on hospital day 11 to a Skilled Nursing Facility to complete the remainder of her antibiotic regimen.

## Discussion

NF is characterized by spreading necrosis of the subcutaneous tissue and fascia that is associated with systemic toxicity and extension along fascial planes.^7^ NF of the breast is rare with a limited number of cases reported to date. More commonly, necrotizing soft tissue infections are described to be found on the extremities, trunk, and perineum. Konik et al.,^8^ Yaji et al.,^9^ Fayman et al.,^2^ Ward et al,^10^ and Shah et al^11^ reported the first cases of breast NF as early as 2001.

The Laboratory Risk Indicator for Necrotizing Fasciitis (LRINEC), first described by Wonget al, is a tool based on six common serum parameters that can be used upon patient presentation to determine risk for NF.^12^ The six serum parameters include C-reactive protein, total white cell count, hemoglobin, serum sodium, creatinine, and glucose. A LRINEC score of 6 or greater suggests a higher risk of NF with a score of 8 or higher having a positive-predictive value of 93.4% for necrotizing soft tissue infection indicating further work-up or emergent operative debridement.^13^ The patient presented in this case report was found to have a LRINEC score of 11 points indicating a high risk for NF. Notably, high clinical suspicion for necrotizing soft tissue infection obviates the usefulness of the LRINEC score with recommendations to instead take the patient for immediate operative debridement.

Despite an initial LRINEC score of 11 points, there was a delay in the management of the patient due to initial misdiagnosis. It was not until hospital day 2 that formal imaging was performed, which was obtained for work-up of abdominal pain rather than for evaluation of the breast abscess. As discussed previously, following a bedside ultrasound of the breast, the surgery team did not suspect NF but rather an infected cyst *vs* an abscess. According to previous literature, NF of the breast can often be misdiagnosed as mastitis, cellulitis, inflammatory breast cancer, or abscess as in this case.^7^ Misdiagnosis of this patient resulted in a delay in operative management for NF. Previously published case reports of breast NF showed that early debridement along with mastectomy was the primary management along with antibiotics.^7^ Mastectomy was the most common treatment among 18 recent cases in a literature review, while the patient presented here underwent two surgical debridements, not requiring mastectomy.^7^

Bedside ultrasound as an adjunct in the work-up for necrotizing soft tissue infection is limited due to lack of resolution of deeper structures, though soft-tissue gas should still be identifiable on ultrasound. Non-specific findings of NF include an echogenic layer of gas above the deep fascia with posterior dirty acoustic shadowing. In addition, hyperechogenicity of the overlying fat can resemble a cobblestone appearance representing subcutaneous edema, however, these findings can also be seen in cellulitis or anasarca. More specific sonographic signs of NF include overall fascia thickening with abnormal fluid collections along fascial planes.^14^

With respect to our patient and her comorbidities, she was at greater risk for developing necrotizing soft tissue infection as she had uncontrolled diabetes mellitus with significantly elevated blood glucose on admission and non-adherence to home diabetes regimen. The patient did report that she had noticed an insect bite to the breast 1 week prior to admission. An insect bite as an inciting event for development of NF is rare in the literature as most cases report trauma to the breast as an etiology (*e.g.* needle core biopsy, mastectomy, breast tumors, etc.).^5,15,16^ Other risk factors, which are not applicable to our present case, include an immunocompromised state, underlying malignancy, current i.v. drug use, chronic renal failure, and peripheral vascular disease. Of note, age is not a risk factor for NF as it can occur at any age despite it being found predominantly in the elderly population.^6^

Once diagnosed, management of NF of the breast includes prompt surgical debridement in combination with broad-spectrum antibiotics. Staged debridements as opposed to immediate mastectomy has become the new standard of practice, however, in most cases of primary NF of the breast, mastectomy is required to achieve adequate source control.^17^ The antibiotic regimen for NF of the breast is broad but usually is narrowed based on the organisms isolated from wound cultures. The infection can be further classified based on the organisms found: Type 1 is considered polymicrobial and can be composed of Gram-positive, Gram-negative, and anaerobes, whereas Type 2 is usually with Group A *Streptococcus* or other beta-hemolytic *Streptococci* in combination with other infectious pathogens.^1,5,18^ Type 2 infections are usually seen with diabetic patients or those with other comorbidities, though our patient, despite being a diabetic, was found to have a wound colonized with *Proteus*, which required a broad-spectrum regimen prior to being narrowed on discharge.

The patient’s new onset AFib that occurred post-debridement was likely secondary to sepsis and undergoing surgery. The patient’s body was overall stressed by her breast NF infection as well as surgical debridement of the wound. AFib is the most common arrhythmia seen in patients with sepsis and hospitalized patients with sepsis have up to a sixfold higher risk of developing atrial fibrillation.^19^ While other cases of breast NF, in the literature to date have not reported AFib as a complication, one case reported that a patient had a myocardial infarction post-operatively as a result of sepsis and multiorgan failure.^2^ Further research and literature reviews are needed to report whether or not cardiac complications such as the ones above may be higher risk with breast NF.

## Conclusions

This case report contributes to the limited literature on breast NF and the management that is necessary for successful treatment. With respect to our patient and her care, we reported a rare case of NF to the breast in a diabetic patient who reported no trauma or recent intervention, but an insect bite prior to admission as the inciting event. Her course was complicated with infection by *Proteus* on wound cultures as well as new onset AFib requiring anticoagulation likely secondary to sepsis and surgical debridement. This emphasizes the importance of careful attention to clinical presentation, imaging, patient medical history, and surgical management in recognizing and treating this rare diagnosis of NF of the breast.

## Learning points

- This case of NF of the breast is rare, with most reports of NF affecting the extremities, abdominal wall, or near the anus or vaginal canal. It is infrequently found within the breast, and most cases present following traumatic events or surgical interventions such as with resection.
- The initial presentation of necrotizing soft tissue infections varies from a minor infection of the soft tissues to severe forms that can present with septic shock or multiorgan system dysfunction and failure. Therefore, early identification of NF is paramount for proper treatment.
- This case emphasizes the importance of careful attention to clinical presentation, imaging, patient medical history, and surgical management in recognizing and treating this rare diagnosis of NF of the breast.
